# A pilot study investigating the relationship between journal impact factor and methodological quality of real-world observational studies

**DOI:** 10.3389/frma.2025.1679842

**Published:** 2025-10-22

**Authors:** Digant Gupta, Amandeep Kaur, Mansi Malik

**Affiliations:** Bridge Medical Consulting Ltd, London, United Kingdom

**Keywords:** impact factor, study quality, Newcastle-Ottawa Scale, critical appraisal, observational study

## Abstract

**Introduction:**

The primary objective of this study was to investigate the association between journal Impact Factor (IF) and study quality in real-world observational studies. The secondary objective was to explore whether the association changes as a function of different study factors (study design, funding type and geographic location).

**Methods:**

Study quality was assessed using the Newcastle-Ottawa Scale (NOS). IFs were obtained from journal websites. The association between journal IF and NOS score was evaluated firstly using Spearman's correlation coefficient, and secondly using one-way Analysis of Variance (ANOVA).

**Results:**

We selected 457 studies published in 208 journals across 11 consecutive systematic literature reviews (SLRs) conducted at our organization over the last 5 years. Most studies were cross-sectional and from North America or Europe. Mean (SD) NOS score was 6.6 (1.03) and mean (*SD*) IF was 5.2 (4.5). Overall, there was a weak positive correlation between NOS score and IF (Spearman's coefficient (ρ) = 0.23 [95% CI: 0.13–0.31]; *p* < 0.001). There was no correlation between NOS score and IF for prospective cohort studies (ρ = 0.07 [95% CI:−0.12–0.25]) and industry-funded studies (ρ = 0.06 [95% CI:−0.09–0.21]). Based on ANOVA, the effect size, eta squared (η^2^), was 0.04 (95% CI: 0.01–0.08), indicating a small effect.

**Discussion:**

While there is some correlation between journal quality and study quality, our findings indicate that high-quality research can be found in journals with lower IF, and assessing study quality requires careful review of study design, methodology, analysis, interpretation, and significance of the findings. Notably, in industry-funded studies, no correlation was found between methodological quality and IF.

## 1 Introduction

An integral part of conducting Systematic Literature Reviews (SLRs) is the quality appraisal of the underlying studies included for reporting (Higgins et al., 2019; Sterne et al., 2019). Quality appraisal is important for multiple reasons. First, it helps to determine whether a study should be included in the SLR. Second, it helps us understand the overall strength of conclusions (e.g., whether any conclusion is based on studies of relatively low quality). Third, it ensures that the interpretation is not distorted by low-quality studies. Finally, it helps when performing sensitivity and subgroup analyses, especially when the SLR is accompanied by a meta-analysis (Deeks et al., 2019).

The journal Impact factor (IF) is the most commonly used indicator for assessing scientific journals. However, it is also frequently used to assess the quality of papers published in a journal. IF is calculated using a specific formula to determine the average number of citations received by articles published in a journal (Ali, 2022; Saha et al., 2003; Waltman and Traag, 2020). One limitation of IF is that while it reflects the average number of citations received by articles in a specific journal, it does not provide information on the actual number of citations of individual papers. Consequently, when IF is used as a proxy for citation frequency, it only accurately evaluates a few papers whose citation counts match the average. Most papers, however, will be misjudged, either being over- or under-evaluated, as their citation numbers will typically fall below or above the average (Abramo et al., 2023; Brito and Rodríguez-Navarro, 2019; Khatoon et al., 2024; Lozano et al., 2012; Waltman and Traag, 2020). Along similar lines, using IF as a proxy for study or research quality might be misleading and is often discouraged (Ali, 2022; Favaloro, 2008; Mason and Singh, 2022; Thelwall et al., 2023; Vakil, 2005).

Typically, our research teams evaluate thousands of observational studies each year using validated quality assessment scales. In the course of our work, we have noted instances of poor-quality studies being published in high-impact journals; and, conversely, high-quality studies being published in low-impact journals.

Upon conducting a comprehensive literature search on this topic, we found limited and inconsistent evidence on the relationship between IF, as a measure of journal “quality,” and the methodological quality of research published in that journal. The evidence is primarily based on data from clinical trials (Ahmed Ali et al., 2017; Gluud et al., 2005; Pinheiro et al., 2024; Saginur et al., 2020) and systematic reviews (Nascimento et al., 2020) or a combination of clinical trials and observational studies (Lee, 2002; Ogihara et al., 2024). While some studies found journal IF to be a poor indicator of study quality (Flint et al., 2019; Nascimento et al., 2020; Ogihara et al., 2024; Saginur et al., 2020), a few reported a weak-to-moderate positive association between the two (Ahmed Ali et al., 2017; Gluud et al., 2005; Lee, 2002; Pinheiro et al., 2024; Thelwall et al., 2023).

The following studies reported journal IF to be a poor indicator of study quality. Across these studies, different measures of association were reported, including correlation coefficients, odds ratios, and regression coefficients. A study by (Ogihara et al., 2024) based on 50 studies (a mix of clinical trials, observational studies, guidelines and reviews) in stroke rehabilitation found a correlation of *r* = 0.235 (*p* = 0.10) between methodological quality score and IF. A study by Flint et al. (2019) based on 72 non-randomized clinical trials of neurocognitive outcomes after pediatric epilepsy surgery found a correlation of *r* = −0.028 (*p* = 0.87) between IF and an average number of common bias sources per study. Saginur et al., analyzing 29 systematic reviews of 189 randomized clinical trials, did not provide correlation coefficients but instead estimated odds ratios with IF >5 as the dependent variable in multivariable model. The analysis showed that the parameters of study quality (such as sample size [aOR (95% CI): 1.01 (0.99, 1.03); *p* = 0.12], randomization of allocation sequence [aOR (95% CI): 1.58 (0.63, 3.93); *p* = 0.33], double blind status [aOR (95% CI): 0.88 (0.21, 3.64); *p* = 0.86], and allocation concealment [aOR (95% CI): 0.53 (0.26, 1.08); *p* = 0.08]) were not statistically significantly associated with journal IF (Saginur et al., 2020). Another study by Nascimento et al. based on 66 systematic reviews of low back pain, likewise, did not report correlation coefficient but reported that journals with higher IF (dependent variable) were not associated with the reviews' methodological quality (independent variable) (ß = −0.3; 95% CI:−4.8, 4.3) (Nascimento et al., 2020).

The following studies reported a weak-to-moderate positive association between IF and study quality. A study by Pinheiro et al. (2024) based on 1,779 randomized clinical trials of physical activity interventions found a correlation of *r* = 0.21 (*p* < 0.001) between trial quality and IF. A study by Thelwall et al. (2023) based on 96,031 studies (all types of studies except review articles) in sciences/arts/humanities found correlations between quality scores and journal impact ranging from *r* = 0.03 to 0.5 across different fields. A study by Lee (2002) based on 243 randomized clinical trials and observational studies in internal medicine found a correlation of *r* = 0.062 (*p* < 0.001) between IF and article quality. Gluud et al. (2005) based on 530 hepatobiliary randomized clinical trials (reported only the *p*-values but no correlation coefficients) found that IF was significantly associated with sample size (*p* < 0.01) and the proportion of trials with adequate allocation sequence generation (*p* < 0.01) or allocation concealment (*p* = 0.02). Finally, a study by Ahmed Ali et al. (2017) based on 750 surgical randomized clinical trials (did not report correlation coefficient) found the presence of sample-size calculation [ß = 1.2; 95% CI: 0.4, 1.9; *p* = 0.002], adequate generation of allocation [ß = 1.0; 95% CI: 0.3, 1.7; *p* = 0.003] and intention-to-treat analysis [ß = 2.0; 95% CI: 1.3, 2.8; *p* < 0.001] to be independently associated with publication in higher IF journals (dependent variable).

A few studies also provided indirect but useful evidence on the relationship between IF and study quality. As an example, one study found that the long-term citation impact of a paper is governed for the most part by the IF of the journal and less so by the quality or the content of the paper, further providing evidence of a relatively weak association between study quality and citedness (Bornmann and Leydesdorff, 2015). On the other hand, an Italian study noted that for some disciplines and under certain circumstances, IF might serve as a useful proxy for the actual quality of an article, potentially being as reliable a predictor as citation counts (Abramo et al., 2010). Similarly, another study using computer simulations showed that the statistical criticisms of using IF to evaluate individual studies are unconvincing, further highlighting inconsistency in the overall evidence base on this topic (Waltman and Traag, 2020). We found no comprehensive studies specifically exploring the association between IF and study quality in the context of real-world observational research.

We hypothesized that journal IF might not necessarily be a good indicator of the methodological quality of published observational research. Consequently, the primary objective of this pilot study was to investigate the association between journal IF and study quality in the context of real-world observational studies. We also hypothesized that the IF-study quality association might be affected by variations in factors such as study design, type of funding and geographic location. Therefore, as a secondary objective, we explored whether the association between journal IF and study quality changes as a function of these factors.

## 2 Materials and methods

### 2.1 Identification and selection of relevant studies

We selected 11 consecutive SLR projects of real-world observational studies conducted by the same research team at our organization over the last 5 years (2019-2023). The following study designs were eligible for inclusion in this study: case-control, cross-sectional, prospective cohort, and retrospective cohort. The SLRs covered a wide range of therapeutic areas including anemia in chronic kidney disease, Angelman syndrome, Crohn's disease, diabetic gastroparesis, diabetic macular edema, diabetic macular ischemia, episodic and chronic migraine, hemophilia, sleep disturbances due to pruritis, treatment-resistant depression, and wet age-related macular degeneration. A consecutive series of projects was chosen to minimize the possibility of selection bias in the identification of studies. Further, this study was restricted to just one research team so as to reduce the inter-team variability in the assessment of study quality.

### 2.2 Study quality and IF assessment

All studies in this analysis were assessed by the same research team, using the Newcastle-Ottawa Scale (NOS) for observational studies (Wells et al., 2012). The NOS contains 3 domains: selection (4 questions), comparability (1 question) and assessment of outcome or exposure (3 questions). Three different NOS instruments were used for cohort studies, case-control studies and cross-sectional studies (the last was an adapted version of the NOS Herzog et al., 2013; refer to Supplementary Table 1 for further details on the three NOS instruments. The scores for all instruments range from a minimum of 0 to a maximum of 9 (with higher ratings indicating better quality). All studies were assessed by one team member, whose results were then verified by a second team member. Any discrepancies were resolved through consensus.

The IFs were obtained directly from the websites of the respective journals. The majority of journals provided IFs for the year 2022; in a few instances, for 2021.

### 2.3 Statistical analysis

In order to inform the choice between parametric and non-parametric statistical procedures, the normality of the NOS score and IF was assessed using the Jarque–Bera (JB) test, defined as JB = n [S^2^/6 + (K-3)^2^/24], where n is the sample size, *S* is skewness, and *K* is kurtosis. The JB statistic is asymptotically distributed as a chi-square distribution with two degrees of freedom, with larger values indicating greater departure from normality (Jarque and Bera, 1987). One-way analysis of variance (ANOVA) or Kruskal-Wallis (K-W) non-parametric test was used to examine the distribution of NOS score and IF across different categories of factors such as study design, geographic location, and type of funding.

The primary objective of the study (i.e., the association between journal IF and NOS score in the overall study sample) was evaluated firstly using Spearman's correlation coefficient and secondly using ANOVA, as described below. Each analytical method provides a different measure of effect size, and together, they allow for a more comprehensive evaluation of the relationship.

Spearman's correlation coefficient, along with 95% CI, was calculated to determine the quantitative relationship between NOS score and journal IF, both for the overall sample (*n* = 457) as well as for different subgroups based on selected stratifying variables (study design, geography, and type of funding). Using Cohen's guidelines, *r* = 0.10, *r* = 0.30, and *r* = 0.50 were considered as cut-offs for small, medium, and large effect sizes, respectively (Cohen, 1992). As part of sensitivity analysis, to assess the possible influence of sampling bias on the results, bootstrap estimation based on 1,000 random samples with replacement was used to generate bias-corrected and accelerated CIs for the correlation coefficient (Bishara and Hittner, 2012). Bootstrapping estimation technique does not assume any level of normally distributed data and therefore tends to be a more robust method for skewed data.

One-way ANOVA was used to examine the mean NOS scores across the 3 categories of IF based on tertiles: low IF ( ≤ 3.2), medium IF (3.3-4.9), and high IF (≥5). The assumption of homogeneity of variance (i.e., variances of NOS scores are equal across IF groups) was assessed using Levene's test. The Brown-Forsythe test and the Welch test were used as robust ANOVA procedures if the homogeneity of variance assumption was not met. The Bonferroni *post-hoc* test (assuming equal variances) or Tamhane's T2 test (assuming unequal variances) were used to explore pairwise differences in mean NOS scores across different IF groups. Eta-squared (η^2^) was calculated as the measure of effect size which indicates the proportion of variation in the NOS score accounted for by the journal IF. Using Cohen's guidelines, the following benchmarks for judging effect size based on η^2^ were used: small (0.01-0.059), medium (0.06-0.139), and large (≥0.14) (Cohen, 2013).

The secondary objective was also analyzed using the same methods, except that the analysis was performed separately within each category of the 3 factors (study design, type of funding, and geographic location). All data were analyzed using SPSS version 28.0 (IBM, Armonk, NY, USA). All analyses were two-tailed, and a difference was considered statistically significant if the *p-* value was < 0.05.

## 3 Results

### 3.1 Evidence base

This pilot study included 457 studies published in 208 unique journals across 11 consecutive SLR projects. As shown in Table 1, the majority of the studies in the sample were cross-sectional, followed by retrospective cohort and prospective cohort studies. With regards to geographical distribution, North America and Europe had the highest representation, followed by Asia-Pacific and multi-region studies. Approximately 40% of the studies were industry-funded. With respect to the underlying disease area, episodic and chronic migraine studies had the highest representation, followed by anemia in chronic kidney disease and sleep disturbances due to pruritis; while diabetic macular ischemia and diabetic gastroparesis were amongst those with the lowest representation. For the overall sample, mean (SD) NOS score was 6.6 (1.03) [median: 7; range 3- 9) and mean (SD) IF was 5.2 (4.5) [median, 3.9; range, 0.2-39).

**Table 1:** Study characteristics.

**Characteristic**	**Categories**	**Number of studies (%) [*N* = 457]**
Study design	Cross-sectional	207 (45.3)
	Retrospective cohort	123 (26.9)
	Prospective cohort	122 (26.7)
	Others^a^	5 (1.1)
Geography	North America	174 (38.1)
	Europe	146 (31.9)
	Asia Pacific	74 (16.2)
	Multi-region^b^	54 (11.8)
	Others^c^	9 (2)
Type of funding	Industry	175 (38.3)
	Non-industry^d^	160 (35)
	Unfunded	50 (10.9)
	Funding undisclosed	72 (15.8)
Disease area	Anemia in chronic kidney disease	56 (12.3)
	Angelman syndrome	37 (8.1)
	Crohn's disease	43 (9.4)
	Diabetic gastroparesis	20 (4.4)
	Diabetic macular edema	39 (8.5)
	Diabetic macular ischemia	23 (5)
	Episodic and chronic migraine	81 (17.7)
	Hemophilia	38 (8.3)
	Sleep disturbances due to pruritis	53 (11.6)
	Treatment-resistant depression	29 (6.3)
	Wet age-related macular degeneration	38 (8.3)

^a^3 case-control, 2, ambispective; ^b^spanning more than 1 continent; ^c^5 from Turkey, 2 from Brazil, 1 from Egypt, 1 from Israel; ^d^Academia, government, non-governmental organization and not-for-profit organization.

Table 2 shows the distribution of NOS score and IF as a function of study characteristics. As a function of *study design*, prospective and retrospective cohort studies had significantly greater NOS scores compared to cross-sectional studies. Also, prospective cohort studies were published in journals with significantly greater IF compared to cross-sectional studies. Based on *geographic location*, North American, European, and multinational studies were published in journals with significantly greater IF compared to studies from Asia Pacific. However, there was no significant association between geographic location and NOS score of a study. Finally, based on *type of funding*, industry-funded and non-industry-funded studies had significantly greater NOS score and IF compared to studies with undisclosed funding.

**Table 2:** NOS score and IF as a function of study characteristics.

	**Mean NOS score**	**ANOVA^a^ *p*-value for NOS**	**Mean rank of IF**	**K-W^b^ *p*-value for IF**
**By study design**				
Cross-sectional	6.33	< 0.001^*^	212.37	0.04^*^
Retrospective cohort	6.98		226.89	
Prospective cohort	6.83		250.07	
**By geography**				
North America	6.77	0.20	251.17	< 0.001^*^
Europe	6.53		228.56	
Asia Pacific	6.58		154.34	
Multinational	6.70		223.73	
**By type of funding**				
Industry	6.80	< 0.001^*^	243.60	< 0.001^*^
Non-industry	6.75		247.16	
Unfunded	6.50		191.01	
Funding undisclosed	6.13		179.53	

ANOVA, Analysis of Variance; IF, Impact Factor; K-W, Kruskal-Wallis; NOS, Newcastle-Ottawa Scale. ^a^For NOS, *S* = −0.292 and excess *K* = −0.032 yielded *JB* = 6.53 with *df* = 2 and *p* = 0.038. Since NOS showed only a borderline deviation from normality, parametric ANOVA was used. ^b^For IF, *S* = 3.68 and excess *K* = 18.69 yielded JB = 7685.14 with df = 2 and *p* < 0.001. Since IF exhibited marked non-normality (strong right skew and heavy tails), non-parametric K-W test was used. The K-W statistic does not test the difference between means but tests the difference between mean ranks. ^*^Significant *p*-values (< 0.05).

### 3.2 Relationship between IF and study quality

**Figure 1:**
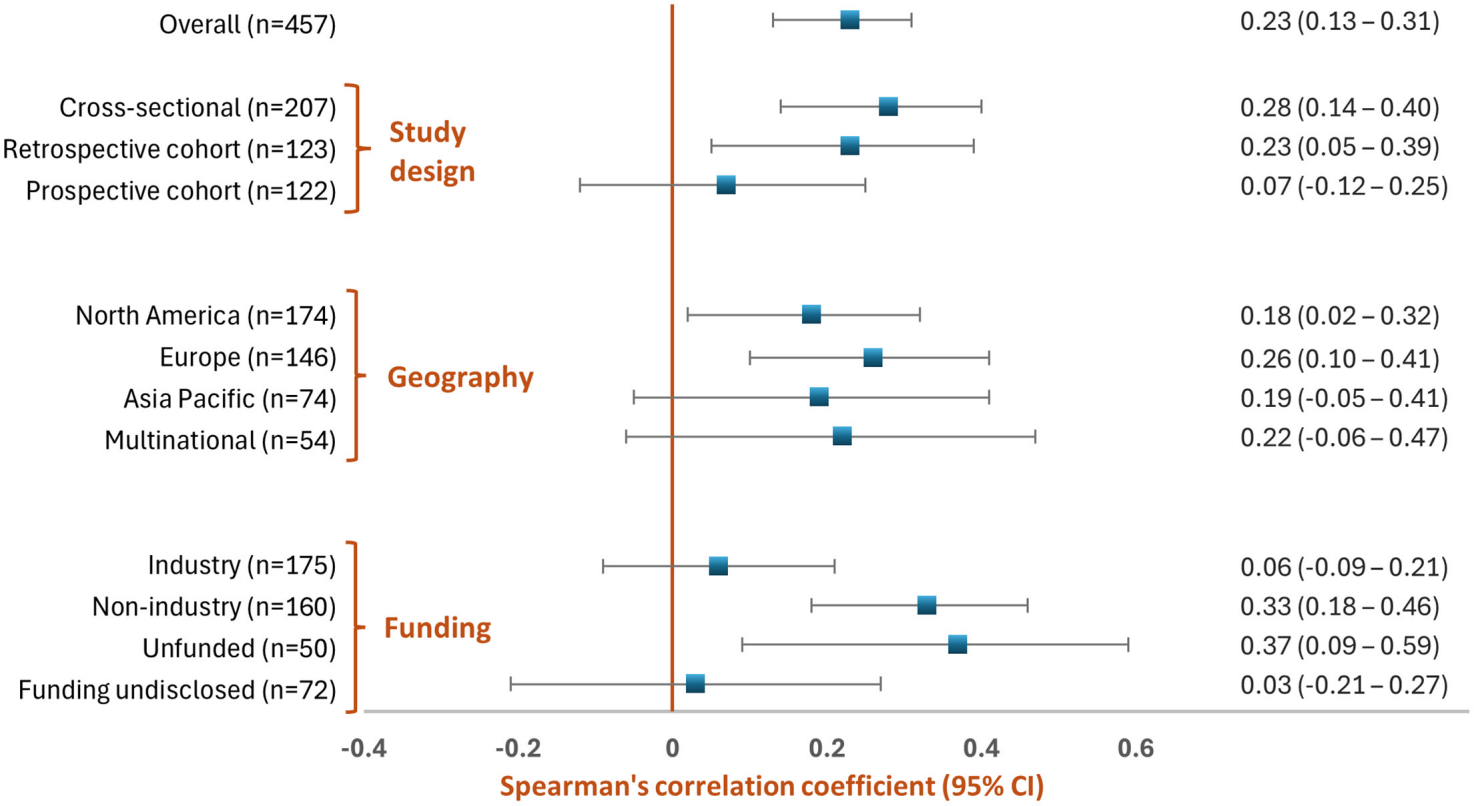
Correlation between IF and NOS score: overall and as a function of study characteristics.

As shown in Figure 1, overall, there was a weak positive correlation between NOS score and IF (Spearman's correlation coefficient (ρ) = 0.23 [95% CI: 0.13–0.31]; *p* < 0.001) for the overall sample of 457 studies. By *study design*, there was a weak positive correlation between NOS score and IF for cross-sectional and retrospective cohort studies whereas there was no correlation between NOS score and IF for prospective cohort studies. By *geographic location*, there was a weak positive correlation between NOS score and IF for all major regions, although only results from North America and Europe were statistically significant (perhaps because of their relatively large sample size). Finally, by *type of funding*, there was a weak positive correlation between NOS score and IF for non-industry funded and unfunded studies, whereas there was no correlation between NOS and IF for industry-funded studies and studies with undisclosed funding.

**Figure 2:**
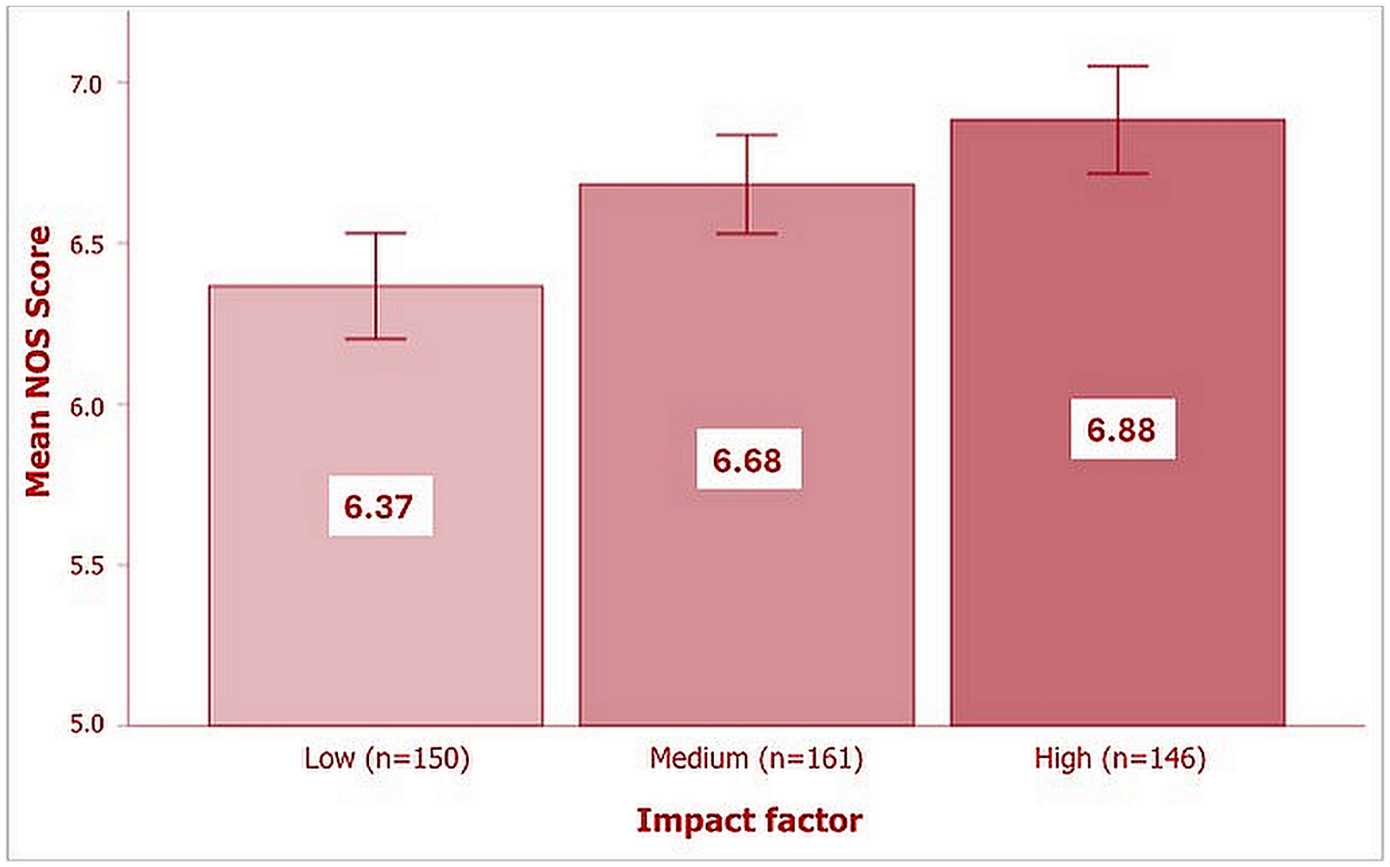
Distribution of NOS score by IF category. The error bars represent 95% CIs of the mean NOS score.

As shown in Figure 2, there was a statistically significant difference in the mean NOS score between the 3 IF groups (low, ≤ 3.2; medium, 3.3-4.9; high, ≥5) as determined by one-way ANOVA [*F*_(2, 454)_ = 9.94, *p* < 0.001]. The effect size, eta squared (η^2^), was 0.04 (95% CI: 0.01-0.08), indicating a small effect (implying that only 4% of variation in the NOS score was accounted for by the journal IF). A *post-hoc* Bonferroni test showed that the mean NOS score was significantly higher in both high IF and medium IF groups compared to the low IF group (*p* < 0.001 and *p* = 0.02 respectively); however, there was no statistically significant difference in the mean NOS score between high and medium IF groups (*p* = 0.25).

## 4 Discussion

We found the overall relationship between IF and NOS score to be positive but weak. Consequently, the IF of a journal is not always a reliable measure of the quality of an individual paper and cannot replace a careful critical appraisal of the underlying research. It is possible that some journals may prioritize novelty over methodological rigor, leading to discrepancies in study quality even among journals with similar IFs. Moreover, journals may be more inclined to publish studies with statistically significant results, leading to publication bias. This can result in high-impact journals publishing studies that are not necessarily of higher quality but are more likely to attract attention and citations. The common theme across these arguments is that the methodological quality of a research paper might be just one of the many factors that journals consider in their editorial decisions. More research is needed to understand what the other factors might be.

The findings of our study are broadly in agreement with those reported by other researchers (Ahmed Ali et al., 2017; Bornmann and Leydesdorff, 2015; Flint et al., 2019; Gluud et al., 2005; Lee, 2002; Nascimento et al., 2020; Ogihara et al., 2024; Pinheiro et al., 2024; Saginur et al., 2020; Thelwall et al., 2023). There remains a fairly consistent suggestion from the existing literature that although there is a positive correlation between expert assessments of article quality and average journal impact across all scientific fields, this correlation is generally weak. Collectively, the majority of the existing body of research along with the findings of the current study suggest that IF should not be used as a proxy for study quality. Even though the correlation between study quality and IF is frequently weak, its generally positive nature makes it tempting for scholars and evaluators to consider IF in their decisions regarding the quality of underlying research, a practice that needs to be discouraged (Thelwall et al., 2023).

Another key finding of our study that warrants some discussion is the lack of correlation between NOS score and IF in industry-funded studies. While it was beyond the scope of this paper to investigate this further, there are several ideas that are worthy of future investigation. For example, are industry-funded studies more likely to report novel and statistically significant findings compared to non-industry-funded studies? How are industry-funded studies perceived by journal editors and by peer reviewers?

We also found a lack of correlation between NOS score and IF for prospective cohort studies. Although this finding needs further evaluation, it is possible that prospective cohort studies, even those of low-to-moderate quality, are likely to attract journals' attention simply by virtue of their design, as a prospective design is inherently associated with a lower risk of bias compared to a case-control or a cross-sectional design. On the other hand, a weak correlation between NOS score and IF for cross-sectional studies suggests that the journal IF is, at least to some degree, indicative of the underlying quality of cross-sectional research.

The strengths of this pilot study include a large sample size of 457 (this is important since, as we have stated, studies of this nature are infrequent in the literature) and that it covers a diverse range of disease areas, making the results more generalizable to the observational research literature. Further, a consecutive series of 11 SLR projects was chosen, reducing selection bias in the identification of studies. The same research team conducted all 11 projects, reducing variability in assessment of quality (members of the team received the same intensive training on critical appraisal using the NOS). Finally, 2 independent researchers scored each research paper during critical appraisal, potentially reducing subjectivity in the assessment.

Some limitations also require acknowledgment. Only one tool (the NOS) was used to assess study quality. Whilst being acknowledged for its ease of use and a convenient scoring system, the NOS has also been criticized for low inter-rater reliability, and its use as a “quantitative” rating scale is not well established (Hartling et al., 2013; Stang, 2010). Again, while IF can provide some insights into the visibility and influence of a journal within its field, it is only one indicator of journal quality. IFs of journals are field-dependent and not comparable across different disease/therapeutic areas (Ahlgren and Waltman, 2014; Bordons et al., 2002; Kurmis, 2003; Owlia et al., 2011; Sajid et al., 2020). As an example, a “top” journal publishing research on rare diseases (i.e., with a very narrow scope) might have an IF lower than the IF of an “average” journal publishing research on a common disease area (i.e., with a broad scope). This study does not allow for causal inferences to be drawn on the relationship between NOS score and IF. Further, the interpretation of effect sizes (Spearman's ρ and eta-squared) is context-dependent, and Cohen's guidelines are not designed to be used to set strict thresholds but rather as a general reference to help interpret the practical significance of findings. The applicability of these findings to the clinical trial literature cannot be assumed. Another limitation of this study is that journal impact factors were obtained directly from the websites of the respective journals rather than from Journal Citation Reports, which is the standard consolidated source. Although the majority of journals reported IFs for 2022 and a few for 2021, this minor year-to-year variation is unlikely to have materially influenced the findings. Finally, the literature review section of this study, while being comprehensive, was not meant to be systematic.

Despite these limitations, the findings of this study have important implications. Clinicians, researchers, and policy makers (and indeed artificial intelligence models) must be trained to critically appraise the methodological quality of an original research paper to make informed decisions, rather than relying on the perceived “prestige” of the journal. Educational initiatives may be needed to help researchers, clinicians, and other stakeholders understand the limitations of journal-based metrics. Journals themselves should consider conducting, and subsequently publishing, a formal quality assessment of studies using a validated tool as part of their peer-review process.

There are several additional avenues for research in this area. Future studies should attempt to confirm these findings by using other tools or checklists for quality assessment of observational studies as well as other indicators of journal quality such as the type of peer review, reputation within the field, editorial policies, and metrics such as SCImago Journal Ranks, Source Normalized Impact per Paper, Article Influence Score, CiteScore, Hirsch Index, and Eigenfactor score (Ali, 2022; Bergstrom, 2007; Owlia et al., 2011; Sajid et al., 2020). Investigating the extent to which publication bias influences the relationship between journal quality and research quality is also important. Finally, qualitative research methods, such as interviews and surveys with researchers, editors, and peer reviewers, can also provide insights into the perceived importance of journal quality and its impact on research practices.

## 5 Conclusion

In summary, while there is some correlation between journal quality and observational study quality, our findings indicate that they are not synonymous. High-quality research can be found in journals with lower IFs and assessing study quality requires careful consideration of study design, methodology, analysis, interpretation, and significance of findings. Notably, in industry-funded studies, no correlation was found between methodological quality and journal IF.

## Data Availability

The raw data supporting the conclusions of this article will be made available by the authors, without undue reservation.
