# Supplementary material for: A pilot study investigating the relationship between journal impact factor and methodological quality of real-world observational studies
Source: Front Res Metr Anal. 2025 Oct 22;10:1679842. doi: 10.3389/frma.2025.1679842 (PMC12586066; doi:10.3389/frma.2025.1679842)
Supplement: Supplementary file 1 [file Table_1.docx]

**Article title:** A pilot study investigating the relationship between journal impact factor and methodological quality of real-world observational studies

**Supplementary Table 1** Newcastle-Ottawa scales by study design

|  | **Cohort** | **Case-Control** | **Cross-sectional** |
| --- | --- | --- | --- |
| **Number of questions/ items** | 8 items | 8 items | 7 items |
| **Domains (number of items)** | **3 domains:**   - Selection (4) - Comparability (1) - Outcome (3) | **3 domains:**   - Selection (4) - Comparability (1) - Exposure (3) | **3 domains:**   - Selection (4) - Comparability (1) - Outcome (2) |
| **Questions (and possible stars)** | **Selection domain**   1. Representativeness of the exposed cohort (*) 2. Selection of the non-exposed cohort (*) 3. Ascertainment of exposure (*) 4. Demonstration that outcome of interest was not present at start of study (*)   **Comparability domain**   1. Comparability of cohorts based on the design or analysis (**)   **Outcome domain**   1. Assessment of outcome (*) 2. Follow-up long enough for outcomes to occur (*) 3. Adequacy of follow-up of cohorts (*) | **Selection domain**   1. Is the case definition adequate? (*) 2. Representativeness of the cases (*) 3. Selection of controls (*) 4. Definition of controls (*)     **Comparability** **domain**   1. Comparability of cases and controls on the basis of the design or analysis (**)   **Exposure domain**   1. Ascertainment of exposure (*) 2. Same method of ascertainment for cases and controls (*) 3. Non-response rate (*) | **Selection domain**   1. Representativeness of the sample (*) 2. Sample size (*) 3. Non-respondents (*) 4. Ascertainment of exposure (**)   **Comparability** **domain**   1. Comparability of different outcome groups based on study design or analysis (**)   **Outcome domain**   1. Assessment of outcome (*) 2. Statistical test (*) |
| **Total stars** | 9 | 9 | 9 |
